# Addressing the carbon footprint, healthfulness, and costs of self-selected diets in the USA: a population-based cross-sectional study

**DOI:** 10.1016/S2542-5196(20)30055-3

**Published:** 2020-03-25

**Authors:** Amelia Willits-Smith, Rodrigo Aranda, Martin C Heller, Donald Rose

**Affiliations:** a School of Public Health & Tropical Medicine, Tulane University, New Orleans, LA, USA; b Center for Sustainable Systems, School for Environment and Sustainability, University of Michigan, Ann Arbor, MI, USA; c Georgia Policy Labs, Georgia State University, Atlanta, GA, USA

## Abstract

**Background:**

The role of diet in health is well established and, in the past decade, more attention has been given to the role of food choices in the environment. The agricultural sector produces about a quarter of the world's greenhouse gas emissions (GHGE), and meat production, especially beef, is an important contributor to global GHGE. Our study aimed to address a fundamental gap in the diet-climate literature: identifying consumers who are receptive to making dietary changes, and the effect of their potential changes on GHGE, diet healthfulness, and diet costs.

**Methods:**

Dietary data on US individuals from a nationally representative survey were linked to food-related GHGE. We identified individuals receptive to changing their diets (potential changers) as those who reported trying US dietary guidance and were likely to agree that humans contribute to climate change. We assessed GHGE, diet healthfulness measured by the Healthy Eating Index (HEI), and diet costs before and after hypothetical changes replacing either beef or meats with poultry or plant-protein foods.

**Findings:**

Our sample comprised 7188 individuals, of whom 16% were potential changers. These were disproportionately women, highly educated, or had higher income compared with individuals deemed not likely to change. Replacing 100% of beef intake in potential changers with poultry reduced mean dietary GHGE by 1·38 kg CO_2_-equivalents per person per day (95% CI 1·19–1·58), a 35·7% decrease. This replacement also increased mean HEI by 1·7% and reduced mean diet costs by 1·7%. We observed the largest changes when replacing all beef, pork, or poultry intake with plant-protein foods (GHGE decreased by 49·6%, mean HEI increased by 8·7%, and dietary costs decreased by 10·5%). Hypothetical replacements in the potential changers alone resulted in whole population reductions in 1-day dietary GHGE of 1·2% to 6·7%, equivalent to 22–126 million fewer passenger vehicle km.

**Interpretation:**

Individual-level diet studies that include a variation in response by consumers can improve our understanding of the effects of climate policies such as those that include sustainability information in national dietary guidance. In our study, we found that changes by a small percentage of motivated individuals can modestly reduce the national dietary GHGE. Moreover, these substitutions can modestly improve diet healthfulness and reduce diet costs for individuals who make these changes.

**Funding:**

Wellcome Trust.

## Introduction

The importance of diet for health has been widely accepted for decades. In the past decade, attention has been focused increasingly on the environmental consequences of food choices, because the footprint of agriculture is large and varies widely between products. The agricultural sector produces about a quarter of the world's greenhouse gas emissions (GHGE).^1^ Animal products typically produce more emissions than plant products. Beef, for example, has among the highest emission levels of all foods, with GHGE per kg about 10 times that of chicken, and about 20 times that of nuts, seeds, or legumes.^2^, ^3^

How can we reduce the carbon footprint of our diets without jeopardising their healthfulness or increasing their costs? This question embodies the triple bottom line of addressing environmental, social, and financial goals. It is also essential in efforts to move towards more sustainable diets, which include environmental, nutrition and health, economic, and sociocultural dimensions.^4^, ^5^

Several studies have used optimisation techniques to show that GHGE can be lowered for diets that still meet nutritional requirements, but these diets were very different from existing eating patterns.^6^, ^7^ Other studies have observed potential reductions in environmental impacts if average national diets shifted towards dietary recommendations.^8^, ^9^ All these studies contributed to our existing knowledge about diet and climate; however, most used aggregate diets, which miss the nuance seen in the wide variation in GHGE of self-selected diets.^10^, ^11^, ^12^ By working with a single datapoint (ie, a national average), these studies were unable to assess which individuals might be receptive to making changes and, ultimately, how policies might be better targeted to induce such changes.

**Research in context**

**Evidence before this study**

The EAT-*Lancet* Commission report proposed a universal healthy reference diet as part of the “Great Food Transformation” necessary to feed the growing global population healthfully while keeping agriculture's impact within the safe operating space of the planet. The proposed diet is substantially different from typical diets from high-income nations, with considerably smaller intakes of animal products and larger intakes of plant-based foods. The Commission emphasised that national commitments are necessary for these shifts, and that all available policy levers should be used. The Dietary Guidelines for Americans (DGA) is one such policy lever that could be used in the USA, which has the second highest level of greenhouse gas emissions (GHGE) globally. The advisory committee recommended inclusion of sustainability as a consideration in the 2015–2020 DGA, but the US government did not follow this recommendation. Expanding the evidence base on the potential effects of policies could facilitate their adoption by policy makers. To survey existing evidence, we searched Web of Science and PubMed for studies published in English up to July 31, 2019, with search terms focusing on the three dimensions of this study: ‘greenhouse gas emissions’, ‘carbon footprint’, and ‘environmental impacts’; ‘diet quality’, ‘nutritional quality’, and ‘diet health’; and ‘diet cost’ and ‘food cost’. We then used backward and forward reference searches for all papers obtained from these reviews and searches. The most recent comprehensive review on this topic found that shifting from typical diets from high-income nations to more sustainable patterns resulted in median GHGE reductions of 20–30%. A previous review found that substitutions could reduce GHGE by up to 50%. Most studies included in these reviews are based on aggregate, rather than individual, dietary data and thus they do not consider variation in population response to policies. Many of these studies also do not have information on whether dietary changes would be affordable and acceptable to consumers. Descriptive research using observed diets indicated that lower-carbon, healthful diets can be about the same cost or cheaper than existing average diets. Development of optimal diets using linear programming has also shown broadly similar findings, but the resulting diets are often complex to communicate.

**Added value of this study**

Our study uses individual dietary intake data to preserve person-to-person variation in food choices and to allow for realistic changes. We assessed not only GHGE changes from different dietary scenarios, but also changes in the nutritional quality of the diet and its cost, addressing the so called triple bottom line of environmental, financial, and social benefits. With microlevel data from a large nationally representative health survey, we were able to model which individuals could be receptive to changing their diets in response to national dietary guidance that incorporates sustainability information. This analysis gives a novel picture of the initial GHGE reductions that are possible when using dietary guidance as a policy lever. Granular data also allow us to design changes that are more realistic, so that changes made by individuals are closest to their existing patterns of eating and are easy to communicate to policy makers and the public.

**Implications of all the available evidence**

Demand-side changes in food systems are an important way to reduce GHGE in high-income countries. However, not all consumers will adopt new behaviours at once. A realistic estimation of the effects of policy change is important for the design and implementation of new policies. Our research indicates that a weak policy lever, such as dietary guidance, would probably affect only a subset of the population, but could still enable some progress towards climate goals while improving nutritional quality and reducing dietary costs.

A growing body of literature exists on the environmental impacts of diets at the individual level.^13^ In 2012, Vieux and colleagues examined various meat reduction and substitution scenarios for French individuals and did not always find GHGE reductions, with results dependent on the food group substituted.^14^ Predefined substitutions for meat were also modelled in a Dutch sample, which showed decreases in GHGE in seven of eight substitutions and reduced mortality risk in all but two.^15^ Additionally, a different Dutch study found decreases in GHGE and some improvements in diet quality when reducing consumption of red and processed meat.^16^ However, none of these individual studies considered environmental impacts, diet quality, and diet cost in a substitution analysis. No individual-level substitution studies have been done in the USA, the country with the second-highest level of GHGE.

To address this gap in the literature, we aimed to examine hypothetical changes in the self-selected diets of individuals in the USA. The focus on individuals allows for a more realistic study, because those receptive to a policy change can be identified. Specifically, we studied the potential effect that inclusion of environmental sustainability in national dietary guidance might have on individuals who are most receptive to following such guidance. After developing an algorithm to identify these individuals, we studied an array of simple substitutions in their diets to assess the potential changes in GHGE, diet quality, and diet cost.

## Methods

### Study population

For this cross-sectional study, we used the 2007–10 waves of the US National Health and Nutrition Examination Survey (NHANES). This is an ongoing nationally representative survey of the US population that is done in 2-year waves and includes modules on demographics, dietary intake, and consumer behaviour. We included in our sample all individuals aged 18–65 years with a reliable dietary intake and with non-missing values on key demographic and behavioural variables. Sociodemographic variables included age, sex, household size, education, income-to-poverty ratio, and race or ethnicity. Income-to-poverty ratio is a measure of household income divided by the poverty guideline. Poverty guidelines, calculated by the US Department of Health and Human Services, are specific to household size, state, and year.^17^ An income-to-poverty ratio lower than 1 means that a household is in poverty. We recoded race or ethnicity into four groups: non-Hispanic white, non-Hispanic black, Hispanic, and other or multiracial. Self-described vegetarian status was determined by asking respondents the following: “do you consider yourself to be a vegetarian?”.

### Diet and emission assessments

Food consumption data in NHANES are based on a 24-h dietary recall, which uses the Automated Multiple-Pass Method that has been described previously.^18^ Our study used data from the day 1 recalls. Reported intake was coded to give consumption of each food item in g for each person. 4623 different food codes were reported in the 2007–10 NHANES. Because most environmental impact data are reported at the level of the commodity, we converted the as-eaten foods reported in NHANES (eg, pepperoni pizza) into consumption of 332 raw commodity ingredients (eg, wheat, milk, pork, and so on) by use of recipe files developed for the Food Commodities Intake Database (FCID).^19^

GHGE were linked to commodity consumption by use of dataFIELD (the database of Food Impacts on the Environment for Linking to Diets). Built through a comprehensive literature review of life cycle assessment studies from 2005 to 2016, dataFIELD includes GHGE values (kg CO_2_-equivalents [CO_2_-eq] per kg of commodity) up to the farm gate for most commodities and to the processor gate for processed commodities such as flours and oils. The FCID recipe files enabled us to adjust for cooked weight so that all impacts are based on as-eaten quantities. Details of the database creation, including a table of food environmental impacts, have been published previously.^2^ The full database can be found online.

The healthfulness of diets was assessed using the Healthy Eating Index 2010 (HEI), a previously validated measure of diet quality.^20^, ^21^ The HEI measures how well a diet corresponds to the Dietary Guidelines for Americans. Scores range from 0 to 100 and include 12 components. Nine components address adequacy (total fruits, whole fruits, total vegetables, dark greens and legumes, whole grains, dairy, total protein foods, seafood and plant proteins, and fatty acid ratio), and three are moderation components, scored higher for lower consumption (refined grains, sodium, and empty calories). We calculated HEI scores for each individual in our NHANES sample by use of an algorithm developed by the National Cancer Institute.^22^ A detailed table of the scoring method is available in the appendix (p 1).

We calculated diet costs for each individual by use of the US Department of Agriculture's Center for Nutrition Policy and Promotion Food Prices database. The database gives the price per 100 g for NHANES’ as-eaten food codes from 2003–04. Updating of the database for 2007–10 NHANES and details of diet cost calculations are described in the appendix (p 2).

### Identifying potential changers

**Figure 1:**
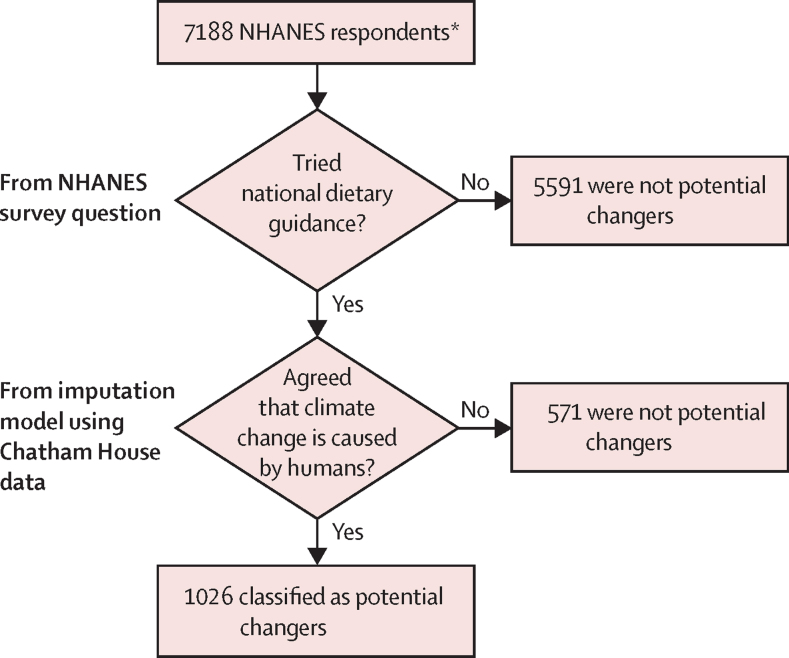
Identification of potential changers We identified potential changers as individuals who might be receptive to changing their diets because of dietary guidance that includes environmental sustainability. NHANES=National Health and Nutrition Examination Survey. *Respondents from 2007 to 2010 with data for dietary guidance items and sociodemographic variables were included.

Which individuals would change their diets if the US government were to include information and suggestions for environmentally sustainable diets in the Dietary Guidelines for Americans? This question was the organising framework for identifying potential changers, who were defined as individuals that tried US dietary guidance and agreed that humans contribute to climate change. The first condition was reported by respondents in NHANES. The second condition was predicted for NHANES respondents by use of answers from similar individuals in another nationally representative survey (figure 1).

We obtained information on NHANES respondents’ previous use of dietary guidance from the Consumer Behaviour Phone Follow-up Module for Adults and coded it into a dichotomous variable. Individuals who answered “yes” to the question “have you tried to follow the (MyPyramid Plan–Pyramid plan) recommended for you?” were coded 1. Individuals who answered “no” to this question and those who said they had not heard of the Food Guide Pyramid or MyPyramid were coded 0.

NHANES does not include questions about attitudes toward climate change, so information on whether an individual agreed that humans contribute to climate change was imputed by use of a survey commissioned by Chatham House and the Glasgow University Media Group.^23^ This Chatham House survey was done online by Ipsos MORI across 12 countries in 2014, and asked a series of questions related to human impacts on climate change. US respondents were coded as a 1 if they answered “strongly agree” or “tend to agree” to the item “to what extent do you agree or disagree with: human activities contribute to climate change.” Other respondents were coded 0.

### Dietary substitutions

We sought to develop a clear set of dietary substitutions that could be communicated easily to policy makers and the public while retaining the complexity of the dietary habits of a diverse population and the nuances in the datasets reflecting those habits. Because of this approach and because NHANES has thousands of foods, we chose general substitutions (eg, poultry for beef) rather than specific ones (eg, “stewed chicken with tomato-based sauce, Mexican style” for “Mexican style beef stew, no potatoes, tomato-based sauce”). We replaced meats in the diet, focusing on beef, because of their effect on the environment.^2^

To implement the substitutions, we operated at the commodity level with use of the FCID. Three replacements were chosen: beef intake replaced with poultry, beef intake replaced with plant-protein foods, and meat intake (beef, pork, and poultry) replaced with plant-protein foods. We used different levels of replacement, in which 100%, 50%, or 25% of the original foods were replaced with the new foods. This range of substitution scenarios, both in terms of amounts substituted and in types of replacements, was selected to provide a full range of possible effects. If an individual did not consume the substituted item (eg, beef) on the interview day, no substitution was made, but the individual was included in the analysis. We limited our analyses to these specific and fixed substitutions and did not investigate complementary changes that consumers might make with reduced beef intake (eg, fewer hamburger buns).

The plant-protein foods used as replacements for meat included 44 individual commodities sorted into three groups: legumes without soy, soy, and nuts and seeds. To make predictions as realistic as possible, replacements accounted for the type and proportions of these foods that individuals were already eating. For example, if a potential changer reported eating only nuts and seeds, but no legumes, then any replacements for meats would be 100% nuts and seeds. For potential changers who did not consume plant-protein foods, mean consumption proportions from the overall sample were used for replacements. A detailed explanation of how these and other substitutions were made on an isocaloric basis is presented in the appendix (pp 3–4).

### Statistical analysis

We used statistical analysis to impute an attitude about climate change to NHANES respondents, to identify differences between potential changers and non-changers, to predict the results of diet substitutions on HEI and diet costs, and to identify differences between baseline and replacement diets.

To impute an attitude about climate change to NHANES respondents, we developed a logistic regression prediction model with the US subsample (n=1051) of the Chatham House data by use of the dichotomous dependent variable (ie, whether an individual agreed or not that humans contribute to climate change) and all independent variables that were also available in NHANES: age, gender, education, household size, and income-to-poverty ratio. We used coefficients from this model and observed demographic characteristics from NHANES respondents to impute the dichotomous attitude variable to NHANES data (appendix pp 5–6, 9).

To identify differences between potential changers and non-changers on demographic variables, we used χ^2^ tests. To identify differences between these groups on meat consumption variables, we used Student's *t* tests.

We examined three outcomes from our diet substitutions: GHGE, HEI, and diet costs; GHGE was calculated, while HEI and diet costs were modelled. We calculated the change in GHGE with replacement diets, because there is a direct linkage from FCID to dataFIELD. However, there is no one-to-one correspondence of FCID commodities to the nutrition-oriented food groups needed for calculation of HEI scores. Therefore, we developed a predictive model of HEI based on aggregate groups of commodities. The model used calculated HEI scores of each individual as the dependent variable and intakes of 19 aggregated commodity groups (eg, beef, poultry, vegetables, and so on) and sociodemographic variables as independent variables. This model was a good predictor of actual HEI (p<0·0001, R^2^ 0·44). Our predictions then used the coefficients from this model with the new food commodity quantities and demographics to predict a post-substitution HEI. The same approach was used for predicting the diet cost for each respondent (p<0·0001, R^2^ 0·32; appendix p 7). The corresponding results are presented as mean differences between baseline and replacement diets, with associated 95% CIs. We used paired *t* tests to test for differences between baseline and replacement diets.

All analyses were done with Stata (version 13) survey procedures, which account for survey design and sampling weights. We used survey strata, primary sampling units, and sampling weights (adjusted for our use of multiple years) available with NHANES data to set up the survey design in a svyset statement. Analyses included mean, tabulate, regress, and logistic procedures with the svy prefix.

### Role of the funding source

The funder of the study had no role in study design, data collection, data analysis, data interpretation, or writing of the report. The corresponding author had full access to all the data in the study and had final responsibility for the decision to submit for publication.

## Results

The total sample size of our study comprised 7188 NHANES respondents. 22% (1597 respondents) of these individuals reported trying dietary guidance, and 69% (4247) were likely to agree that human activities contribute to climate change. Of these, 16% (1026) were in both groups, and thus were classified as potential changers. The overall sample reflected the US population: women comprised slightly over half of participants and about two-thirds of participants were non-Hispanic white (table 1). Only 2% (164) of 7188 individuals were self-described vegetarians.

**Table 1:** NHANES sample characteristics*

		**Full sample (n=7188)**	**Not potential changer (n=6162)**	**Potential changer**†**(n=1026)**	**p value**‡
Sex		..	..	..	<0·0001
	Men	3360 (47%; 46–49)	3139 (52%; 50–54)	221 (22%; 19–26)	..
	Women	3828 (53%; 51–54)	3023 (48%; 46–50)	805 (78%; 75–81)	..
Age (years)		..	..	..	0·10
	18 to 29	1751 (24%; 22–26)	1515 (24%; 22–27)	236 (22%; 19–26)	..
	30 to 49	3051 (44%; 42–46)	2613 (45%; 42–47)	438 (42%; 38–46)	..
	50 to 65	2386 (32%; 30–34)	2034 (31%; 29–33)	352 (36%; 32–40)	..
Race or ethnicity		..	..	..	<0·0001
	Non-Hispanic white	3208 (69%; 64–74)	2677 (68%; 63–73)	531 (75%; 69–80)	..
	Non-Hispanic black	1477 (12%; 10–14)	1258 (12%; 10–14)	219 (11%; 9–14)	..
	Hispanic	2187 (14%; 11–18)	1952 (15%; 11–19)	235 (10%; 7–14)	..
	Other	316 (5%; 5–7)	275 (6%; 5–7)	41 (5%; 3–6)	..
Education		..	..	..	<0·0001
	Lower than high school	1875 (17%; 16–19)	1781 (19%; 17–21)	94 (7%; 5–11)	..
	High-school graduate or equivalent	1695 (24%; 22–26)	1514 (25%; 23–27)	181 (16%; 13–20)	..
	Some college	2141 (31%; 30–33)	1707 (30%; 28–31)	434 (39%; 35–43)	..
	College graduate or higher	1477 (28%; 26–31)	1160 (26%; 24–29)	317 (37%; 33–42)	..
Income-to-poverty ratio		..	..	..	<0·0001
	<1	1336 (12%; 10–14)	1209 (13%; 11–15)	127 (7%; 6–9)	..
	1 to <2	1928 (18%; 17–20)	1765 (20%; 18–21)	163 (11%; 8–15)	..
	2 to <5	2434 (38%; 36–41)	2000 (37%; 35–40)	434 (42%; 38–45)	..
	≥5	1490 (32%; 29–35)	1188 (30%; 27–33)	302 (40%; 35–46)	..
Self-described vegetarian		164 (2%; 2–3)	131 (2%; 1–2)	33 (4%; 2–6)	0·02
Beef consumption (g/day)§		51·3 (47·7–54·8)	53·9 (50·2–57·6)	37·7 (32·3–43·0)	<0·0001
Pork consumption (g/day)§		29·1 (26·8–31·5)	30·9 (28·5–33·3)	20·1 (15·8–24·5)	<0·0001
Poultry consumption (g/day)§		55·9 (52·4–59·4)	56·3 (52·3–60·3)	53·8 (49·9–57·7)	0·35

Data are n (%; 95% CI) or mean (95% CI); all analyses account for survey design and sampling weights. NHANES=US National Health and Nutrition Examination Survey.

* NHANES 2007–2010 adults (aged 18–65 years) who responded to questions about trying dietary guidance (MyPyramid or Food Guide Pyramid).

† Potential changers are individuals who reported trying dietary guidance and were estimated to be likely to agree that humans contribute to climate change; these individuals comprised 16% (95% CI 15–17) of the sample.

‡ We used χ^2^ tests to test for association between being a potential changer (or not) and each of the categorical demographic variables; we used Student's *t* test to test for differences between potential changers and non-changers on each of the consumption variables.

§ Commodity amounts of edible portion of the meats.

Potential changers were more likely to be women, to be more highly educated, or to have higher income than those classified as non-changers. Compared with non-Hispanic white individuals, those who were of other ethnicities were less likely to be potential changers. We observed no significant differences in age between potential changers and the rest of the sample. Potential changers were more likely to describe themselves as vegetarians and, on average, consumed less beef and pork (but not poultry) than other respondents.

Baseline mean dietary GHGE among potential changers was 3·88 kg CO_2_-eq per person per day (95% CI 3·64–4·12; table 2). Beef replacement predictions were run on the 61% (645) potential changers who reported eating beef on their dietary recall day, which amounted to 10% of the overall sample. In potential changers, replacing 100% of beef intake with poultry reduced mean dietary GHGE by 1**·**38 kg CO_2_-eq per person per day (95% CI 1·19–1·58; table 2). This change also increased the mean estimated HEI and decreased mean estimated dietary costs, representing a decrease in mean GHGE of 35·7%, a 1·7% increase in mean HEI, and a 1·7% decrease in mean cost (table 2). Replacing the beef with plant-protein foods reduced the mean GHGE of potential changers by 40·3%, increased mean HEI by 3·3%, and decreased mean dietary cost by 5·5%. Replacing less than 100% of beef intake in the diet of potential changers resulted in similar, but smaller, modifications in GHGE, HEI, and diet costs. Absolute quantities of meats and plant proteins before and after replacements are presented in the appendix (p 8).

**Table 2:** Results of hypothetical meat reductions among potential changers in dietary GHGE, Healthy Eating Index, and dietary costs

		**Food-related GHGE (kg CO**_2_-equivalents per person per day)*			Estimated Healthy Eating Index*			Estimated diet cost (US$ per person per day)*		
		Mean	Mean change†	Change (%)‡	Mean	Mean change†	Change (%)‡	Mean	Mean change†	Change (%)‡
Original diet		3·88 (3·64 to 4·12)	..	..	52·65 (51·97 to 53·32)	..	..	5·24 (5·16 to 5·32)	..	..
100% beef replaced										
	With poultry	2·50 (2·36 to 2·64)	−1·38 (−1·58 to −1·19)	−35·7	53·53 (52·82 to 54·24)	0·88 (0·76 to 1·00)	1·7	5·15 (5·08 to 5·22)	−0·09 (−0·10 to −0·08)	−1·7
	With plant protein§	2·32 (2·18 to 2·46)	−1·56 (−1·79 to −1·34)	−40·3	54·39 (53·64 to 55·14)	1·74 (1·40 to 2·08)	3·3	4·95 (4·87 to 5·03)	−0·29 (−0·33 to −0·25)	−5·5
50% beef replaced										
	With poultry	3·19 (3·03 to 3·35)	−0·69 (−0·79 to −0·59)	−17·8	53·09 (52·40 to 53·77)	0·44 (0·38 to 0·50)	0·8	5·20 (5·12 to 5·27)	−0·05 (−0·05 to −0·04)	−0·9
	With plant protein§	3·10 (2·94 to 3·26)	−0·78 (−0·89 to −0·67)	−20·1	53·52 (52·82 to 54·22)	0·87 (0·70 to 1·04)	1·7	5·10 (5·02 to 5·17)	−0·14 (−0·17 to −0·12)	−2·8
25% beef replaced										
	With poultry	3·54 (3·34 to 3·74)	−0·35 (−0·40 to −0·30)	−8·9	52·87 (52·19 to 53·55)	0·22 (0·19 to 0·25)	0·4	5·22 (5·14 to 5·29)	−0·02 (−0·03 to −0·02)	−0·4
	With plant protein§	3·49 (3·29 to 3·69)	−0·39 (−0·45 to −0·34)	−10·1	53·08 (52·40 to 53·77)	0·44 (0·35 to 0·52)	0·8	5·17 (5·09 to 5·25)	−0·07 (−0·08 to −0·06)	−1·4
100% beef, pork, or poultry replaced with plant protein§		1·96 (1·84 to 2·08)	−1·93 (−2·14 to −1·71)	−49·6	57·22 (56·20 to 58·23)	4·57 (4·04 to 5·09)	8·7	4·69 (4·56 to 4·81)	−0·55 (−0·59 to −0·51)	−10·5
50% beef, pork, or poultry replaced with plant protein§		2·92 (2·76 to 3·08)	−0·96 (−1·07 to −0·86)	−24·8	54·93 (54·15 to 55·71)	2·28 (2·02 to 2·55)	4·3	4·96 (4·87 to 5·06)	−0·28 (−0·30 to −0·26)	−5·3
25% beef, pork, or poultry replaced with plant protein§		3·41 (3·21 to 3·61)	−0·48 (−0·54 to −0·43)	−12·1	53·79 (53·08 to 54·50)	1·14 (1·01 to 1·27)	2·2	5·10 (5·02 to 5·19)	−0·14 (−0·15 to −0·13)	−2·6

Data are mean (95% CI); all analyses account for survey design and sampling weights. Potential changers (n=1026) are individuals who reported trying dietary guidance and were estimated to be likely to agree that humans contribute to climate change; these individuals comprised 16% (95% CI 15–17) of the sample. All replacements were made in equal calorie amounts, as estimated from the National Nutrient Database for Standard Reference. Replacements were only made if individuals consumed the meats in question: 645 (61%) ate beef and 938 (92%) ate beef, pork, or poultry; however, mean changes included all potential changers, whether a replacement was made or not. GHGE=greenhouse gas emissions. NHANES=US National Health and Nutrition Examination Survey.

* Food-related GHGE were calculated on the basis of commodity intakes by use of the database of Food Impacts on the Environment for Linking to Diets; mean results are based on calculations of substitutions at the individual level, with variability due to sampling error in NHANES; HEI and diet cost results are means of person-level predicted values and associated CIs; predictions were based on commodity intakes and sociodemographic variables (appendix p 5).

† A paired *t* test was used to test the hypothesis that the mean difference between individuals' substituted diets and original diets was equal to 0; all differences in the table were significant at a level of p<0·0001.

‡ Values are percent change in the mean value compared with that of baseline (original).

§ Plant proteins are legumes, nuts, and seeds; diet changes for each potential changer reflected the individual's actual reported intakes of these three food groups; replacements were made in the same ratio as that the individual reported eating the three food groups; if the individual did not eat any of the food groups, the overall average ratio in the sample was used to distribute the new intake, specifically 0·405 for legumes other than soy, 0·336 for nuts or seeds, and 0·259 for soy.

Almost all (92%; n=938) potential changers ate beef, pork, or poultry on their recall day, therefore, when substituting for these meats, we ran scenarios on 15% of the overall sample. Replacing 100% of beef, pork, and poultry intake in the diet of these potential changers with plant-protein foods lowered mean GHGE by 49·6%, increased mean HEI by 8·7%, and decreased dietary cost by 10·5%. Replacing only a quarter of this meat intake in the diet of potential changers reduced mean GHGE by 12·1%, increased mean HEI by 2·2%, and decreased cost by 2·6% (table 2).

We assessed the effect of these substitutions made by potential changers on the overall sample (table 3). Replacing 100% of beef with either poultry or plant-protein foods in the diet of only the potential changers reduced the mean GHGE in the overall sample by about 5%. Replacing meat and poultry with plant proteins lowered GHGE by 6·7%.

**Table 3:** Total US food-related GHGE after hypothetical changes in meat intake among potential changers

		**Mean per person per day (n=7188)**			Population-level impact per day*		
		Mean (kg CO_2_-eq)	Mean change (kg CO_2_-eq)†	Change (%)‡	Total (metric tonnes CO_2_-eq)	Change in total (metric tonnes CO_2_-eq)	Equivalent difference in passenger vehicle km§
Original diet		4·64 (4·48 to 4·80)	..	..	475 410	..	..
100% beef replaced							
	With poultry	4·41 (4·27 to 4·55)	−0·22 (−0·26 to −0·19)	−4·8	452 471	−22 939	−90 484 398
	With plant protein¶	4·39 (4·25 to 4·53)	−0·25 (−0·29 to −0·21)	−5·4	449 504	−25 906	−102 187 126
50% beef replaced							
	With poultry	4·53 (4·39 to 4·67)	−0·11 (−0·13 to −0·09)	−2·4	463 941	−11 469	−45 242 199
	With plant protein¶	4·51 (4·37 to 4·65)	−0·13 (−0·15 to −0·11)	−2·7	462 457	−12 953	−51 093 563
25% beef replaced							
	With poultry	4·58 (4·46 to 4·70)	−0·06 (−0·07 to −0·05)	−1·2	469 676	−5734	−22 621 099
	With plant protein¶	4·56 (4·42 to 4·70)	−0·06 (−0·07 to −0·05)	−1·4	468 934	−6476	−25 546 782
100% beef, pork, or poultry replaced with plant protein¶		4·33 (4·19 to 4·47)	−0·31 (−0·35 to −0·27)	−6·7	443 494	−31 916	−125 891 484
50% beef, pork, or poultry replaced with plant protein¶		4·48 (4·34 to 4·62)	−0·16 (−0·18 to −0·13)	−3·4	459 452	−15 958	−62 945 742
25% beef, pork, or poultry replaced with plant protein¶		4·56 (4·42 to 4·70)	−0·08 (−0·09 to −0·07)	−1·6	467 431	−7979	−31 472 871

Data are mean (95% CI), unless specified otherwise; all analyses account for survey design and sampling weights. Potential changers (n=1026) are individuals who reported trying dietary guidance and were estimated to be likely to agree that humans contribute to climate change; these individuals comprised 16% (95% CI 15–17) of the sample. All replacements were made in equal calorie amounts, as estimated from the National Nutrient Database for Standard Reference. Replacements were only made if individuals consumed the meats in question: 645 (61%) ate beef and 938 (92%) ate beef, pork, or poultry. GHGE=greenhouse gas emissions. NHANES=US National Health and Nutrition Examination Survey. CO_2_-eq=CO_2_-equivalents.

* Population-level values were calculated by use of the probability weights (expansion factors) supplied with the NHANES dataset; these represent the size of the population at the midpoint of the survey years being used; in the case of NHANES 2007–10, this was 153 731 402 individuals.

† A paired *t* test was used to test the hypothesis that the mean difference between individuals' substituted diets and original diets was equal to 0; all differences in the table were significant at a level of p<0·0001.

‡ Values are percent change in the mean value compared with that of baseline (original).

§ Calculated with the US Environmental Protection Agency's Greenhouse Gas Equivalencies Calculator.

¶ Plant proteins are legumes, nuts, and seeds; diet changes for each potential changer reflected the individual's actual reported intakes of these three food groups; replacements were made in the same ratio as that the individual reported eating the three food groups; if the individual did not eat any of the food groups, the overall average ratio in the sample was used to distribute the new intake, specifically 0·405 for legumes other than soy, 0·336 for nuts or seeds, and 0·259 for soy.

**Figure 2:**
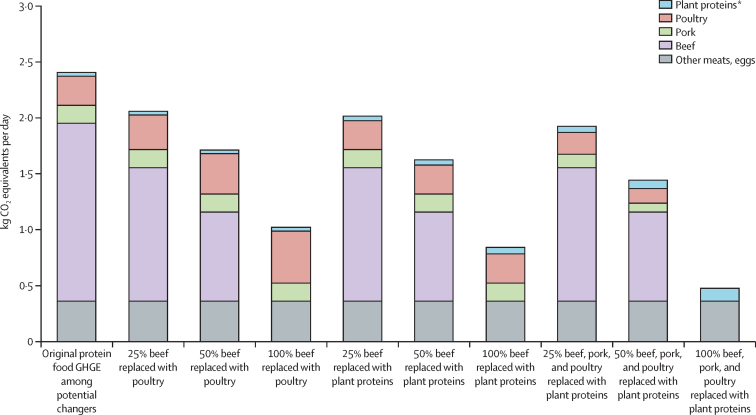
GHGE from protein foods in potential changers before and after hypothetical changes Data are mean (95% CI); all analyses account for survey design and sampling weights. Potential changers (n=1026) were individuals who reported trying dietary guidance and were estimated to be likely to agree that humans contribute to climate change. These individuals were 16% of the sample (95% CI 15–17). All replacements were made in equal calorie amounts, as estimated from the National Nutrient Database for Standard Reference. Replacements were only made if individuals consumed the meats in question: 645 (61%) ate beef, and 938 (92%) ate beef, pork, or poultry. GHGE=greenhouse gas emissions. *Plant proteins are legumes, nuts, and seeds; diet changes for each potential changer reflected the individual's actual reported intakes of these three food groups; replacements were made in the same ratio as that the individual reported eating the three food groups; if the individual did not eat any of the food groups, the overall average proportions in the sample were used to distribute the new intake, specifically 0·405 for legumes other than soy, 0·336 for nuts or seeds, and 0·259 for soy.

We also assessed how changes in intakes from the different protein food groups contributed to GHGE reductions among potential changers (figure 2). Beef intake represented most GHGE from the original diet and remained the largest share of GHGE after any substitution scenario except 100% beef replacement. In other words, beef was still the largest contributor to emissions even when intake was reduced.

## Discussion

Replacing beef or beef, pork, and poultry in the diets of motivated consumers reduced the GHGE associated with their diets by an average of 9% to 50% depending on the type and degree of substitution. Although these environmental impacts were substantial among potential changers, because they comprised only 16% of the sample (1026 respondents), overall dietary GHGE changes at the population level were much smaller. Diet changes also increased the healthfulness of the diets in potential changers and reduced diet costs. In general, diet quality in the USA (as measured by HEI) is low and the modest changes that we observed in this study are a step in the right direction. Although the greatest reductions in emissions came from substituting plant-protein foods for all beef, pork, and poultry intake, replacing just the beef intake would account for more than 80% of this reduction.

Our GHGE results are broadly consistent with previous research. For example, in a review of studies on the environmental impacts of dietary change, Aleksandrowicz and colleagues found decreases in GHGE of 3% to 36% when meat from ruminant animals (eg, beef or lamb) was replaced with meat from monogastric animals (eg, chicken or pork), and decreases of 15% to 58% with changes to vegetarian diets.^24^ In our study, complete substitution of beef for poultry resulted in a decreased GHGE of 35·7% for potential changers, whereas shifting away from all meats to vegetarian protein foods resulted in a 49·6% drop. Substantial reductions in meat intakes are also recommended by expert committee reports.^25^, ^26^

Our results on diet healthfulness and cost are also consistent with the literature. Most, but not all, studies have shown that positive improvements in diet healthfulness are concomitant with reductions in GHGE. For example, of the 37 scenarios in the review by Aleksandrowicz and colleagues that modified diets to meet health guidelines, only four had an increase in GHGE.^24^ All scenarios in our study both reduced GHGE and improved diet healthfulness. Optimisation studies have shown that healthier and more sustainable diets can be obtained for modest reductions in cost (3–11%), which was similar to our results.^6^, ^27^

These comparisons are based on our results for potential changers. Because they accounted for only 16% of the sample, our overall population estimates for GHGE reductions are much smaller than those of other studies. This is probably a more realistic short-term outcome for attempts to move towards more climate-friendly diets, because the entire population is not expected to make immediate changes. Still, considering the scale of these food replacements relative to other GHGE in the USA, our diet scenarios produced reductions equivalent to 22–126 million fewer km driven in a passenger vehicle for each day of intake.^28^

Clearly, the transition to climate-friendly diets will require new research and new policy work. We used a relatively weak policy lever—information from dietary guidance—to motivate change.^29^, ^30^ We assumed that individuals who had tried to follow guidance before and who agreed that humans caused climate change would make changes to their diets to reduce their carbon footprint, if such information was newly included in dietary guidelines. However, food choice behaviour is complex and multi-faceted.^31^ Taste, cultural preferences, convenience, and costs are all important factors that shape this behaviour, in addition to health and environmental concerns. Moreover, several relevant aspects of meat-eating behaviour have been documented, such as consumer attachment to eating meat and various rationalisations for it.^32^, ^33^, ^34^ The potential changers we identified already consumed less beef and pork at baseline than other individuals in the sample, but, unfortunately, we don't know how attached they might be to eating beef. Therefore, how much they would reduce their beef intake is an open question, which is why we assessed several scenarios. We were not able to better model consumption decisions, either who would make diet changes or by how much, because NHANES and other nationally representative surveys in the USA do not include questions on attitudes or behaviours towards climate action or meat consumption. Future research would benefit from such an instrument.

Our study had other limitations. We used a static analysis focused only on specific fixed diet modifications of potential changers. We did not investigate other dietary changes that might accompany this reduction of meats or secondary effects on production, market supply, beef prices, or consumption of non-changers, either within the USA or internationally. For example, the reductions in GHGE described here could be muted if excess supply is shifted overseas. As such, our estimates are better thought of as potential first-order, short-run changes. Finally, food production has other environmental impacts, such as land and water use, which could be modelled in the future.^35^

An overall strength of our study is the realistic nature of the dietary changes. Changes were made only in the portion of the population that was more likely to be motivated by this policy lever. We included modest change scenarios that avoided the complete elimination of food groups. Changes in food groups (amounts of reductions in meat and increases in poultry, legumes, nuts, or seeds) were based on how much individuals were already eating, and replacements took into consideration the proportions in which they ate different commodities within these groups. These choices minimised the differences from the existing diets of potential changers, making them more likely to be acceptable to consumers. Another strength of this study was the underlying dataset developed for it. The GHGE values came from a comprehensive approach to match detailed food consumption data with the latest literature on environmental impacts.

In conclusion, changes in food consumption by a small percentage of motivated individuals can reduce food-related GHGE, increase diet healthfulness, and reduce diet costs. These changes in motivated consumers can have an effect, albeit modest, on emissions at the national level. Our study provides additional evidence that it is worthwhile to provide environmental sustainability as well as nutrition information to US consumers. While dietary guidance policy is one way to disseminate this information, other methods should also be considered.
