# Supplementary material for: Addressing the carbon footprint, healthfulness, and costs of self-selected diets in the USA: a population-based cross-sectional study
Source: Lancet Planet Health. 2020 Mar 25;4(3):e98–e106. doi: 10.1016/S2542-5196(20)30055-3 (PMC7232940; doi:10.1016/S2542-5196(20)30055-3)
Supplement: Supplementary appendix [file mmc1.pdf]

THE LANCET
Planetary Health

## Supplementary appendix

This appendix formed part of the original submission and has been peer reviewed. We post it as supplied by the authors.

Supplement to: Willits-Smith A, Aranda R, Heller M C, et al. Addressing the carbon footprint, healthfulness, and costs of self-selected diets in the USA: a population-based cross-sectional study. *Lancet Planet Health* 2020: **4:** e98–106.

# Supplementary Appendix

## Appendix Table 1. Point allocation in the 2010 Healthy Eating Index (HEI)*

| | Dietary component | Maximum Points† | Standard for maximum score‡ | Standard for minimum score (zero) |
|---|---|---|---|---|
| Adequacy§ | Total Fruit | 5 | ≥ 0·8 c-eq/1000 kcal | No fruit |
| | Whole Fruit | 5 | ≥ 0·4 c-eq/1000 kcal | No whole fruit |
| | Total Vegetables | 5 | ≥ 1·1 c-eq/1000 kcal | No vegetables |
| | Dark Greens and Legumes | 5 | ≥ 0·2 c-eq/1000 kcal | No dark-green vegies, beans, or peas |
| | Whole Grains | 10 | ≥ 1·5 oz-eq/1000 kcal | No whole grains |
| | Dairy | 10 | ≥ 1·3 c-eq/1000 kcal | No dairy |
| | Total Protein Foods | 5 | ≥ 2·5 oz-eq/1000 kcal | No protein foods |
| | Seafood and Plant Proteins | 5 | ≥ 0·8 c-eq/1000 kcal | No seafood or plant proteins |
| | Fatty Acid Ratio¶ | 10 | (PUFAs+MUFAs)/SFAs ≥ 2·5 | (PUFAs+MUFAs)/SFAs ≤ 1·2 |
| Moderation** | Refined Grains | 10 | ≤ 1·8 oz-eq/1000 kcal | ≥ 4·3 oz-eq/1000 kcal |
| | Sodium | 10 | ≤ 1·1 g/1000 kcal | ≥ 2·0 g/1000 kcal |
| | Empty Calories | 20 | ≤ 19% of energy | ≥ 50% of energy |

* Source.[1]
† Points are allocated based on linear interpolation between specific densities (quantity/1000 kcal) or ratios that are assigned for minimum (zero) and maximum scores.
‡ Abbreviations: c-eq = cup equivalent; kcal = kilocalories; oz-eq = ounce equivalent; g = grams
§ Higher score indicates higher consumption.
¶ Ratio of poly- and mono-unsaturated fatty acids to saturated fatty acids.
** Higher score indicates lower consumption.

**Diet Cost Calculations**

Diet cost was calculated for each individual using USDA's Center for Nutrition Policy and Promotion Food Prices Database. This represented 72% (n=4,251) of foods needed for the 2007-2010 NHANES. Food codes that were new in 2007-2010 were given the price of a similar item from the earlier time period. For example, “Buttermilk, fluid, whole” was matched with the price for “Buttermilk, fluid, 2% fat.” Alcoholic beverages were not included in the CNPP database. Prices for these were calculated using the USDA Economic Research Service Quarterly Food-Away-From-Home Prices (which includes food-at-home prices for alcoholic beverages) and recipes developed from the Food and Nutrient Database for Dietary Studies. All foods were adjusted for inflation to 2009-2010 US dollars using the Consumer Price Index from the Bureau of Labor Statistics. Diet cost for individuals was calculated by multiplying grams of intake by price per gram and adding them for the day. The CNPP database prices are for food-at-home. Eating away from home is common in the USA, and as such, the diet costs calculated here likely underestimate true costs.

**Isocaloric replacement of meats**

All substitutions employed in this research were isocaloric. Averages from the National Nutrient Database for Standard Reference (SR28) were used to create conversion factors for all necessary replacements. For example, the mean energy content in 100 grams of raw beef was 188 kilocalories (kcal) and for poultry the value was 168 kcal. Therefore, replacements of beef with poultry used a conversion of 1·12 to scale up the amount of poultry to the same energy value as the beef it replaced.

Nutrient content from all analytic entries of the following food groups was extracted from the National Nutrient Database for Standard Reference (SR28): beef products, pork products, poultry products, legumes and legume products, and nut and seed products. "Composite" items such as "Beef, composite of trimmed retail cuts, separable lean and fat, trimmed to 0" fat, all grades, raw," are aggregations of other SR28 entries. As such, these were excluded. Items missing nutrient information were also excluded (5 cured beef items). Coconut entries were excluded from the nuts and seeds category in order to align with commodity groupings from FCID. Peanuts were included as nuts, not legumes. The legumes group in the SR28 included soy products; we separated these to better match FCID commodities. Of the available soy items in the SR28, those that could be commonly used as plant-based protein sources were included (e.g. tofu and soymilks). Highly processed entries such as soy creamers were excluded.

Mean kilocalories per 100 grams were calculated for all categories: raw beef products, raw pork products, raw poultry products, raw legumes, soy, and raw nuts and seeds. See Appendix Table 2.

**Appendix Table 2. Mean Energy Content per 100 grams in SR28 entries**

| | **Kilocalories** | **95% CI** | **Count** |
|---|---|---|---|
| | 188·1 | (179·1, 197·1) | 408 |
| Raw pork products | 209·1 | (177·5, 240·8) | 95 |
| Raw poultry products | 167·7 | (154·3, 181·1) | 154 |
| Raw legume products, excluding soy | 344·7 | (339·1, 350·2) | 33 |
| Soy products, excluding highly processed | 120·2 | (95·5, 144·9) | 91 |
| Raw nuts and seeds | 479·2 | (413·9, 544·6) | 34 |

**Isocaloric replacement of meats (continued)**

To calculate conversion factors for replacements, the average nutrient value in the food to be replaced was divided by the average nutrient value in the replacement food. For example, the mean energy content in 100 grams of raw beef was 188 kilocalories (kcal) and for poultry the value was 168 kcal. Therefore, replacements of beef with poultry used a conversion of 1·12 to scale up the amount of poultry to the same energy value as the beef it replaced. Appendix Table 3 shows conversion factors for isocaloric replacements for all food groups.

**Appendix Table 3. Isocaloric substitution factors**
(grams needed to replace 1 gram of the column food)

| | Beef | Pork | Poultry |
|---|---|---|---|
| Poultry | 1·12 | ·· | ·· |
| Legumes, excluding soy | 0·55 | 0·61 | 0·49 |
| Nuts and seeds | 0·39 | 0·44 | 0·35 |
| Soy | 1·57 | 1·74 | 1·39 |

For changes including legumes, nuts, and seeds replacements, the following calculations were done at the individual level. The grams of beef (or of beef, of poultry, and of pork) to be replaced was calculated. For example, if the person ate 80 grams of beef, 40 grams would need to be replaced under the 50% scenario. Those 40 grams would be allocated among the legumes, soy, and nuts/seeds categories according to the ratios in which that person ate those foods. For the 8% of changers who did not consume any of these foods (n=106), their replacement grams were allocated at the overall ratios for the population: 0·405 legumes other than soy, 0·336 nuts/seeds, and 0·259 soy. To replace the 40 grams of beef using these proportions, for example, the amounts would be as follows:

- Legumes other than soy: 40 grams * 0·405 * 0·55 = 8·91 grams
- Nuts/seeds: 40 grams * 0·336 * 0·39 = 5·24 grams
- Soy: 40 grams * 0·259 * 1·57 = 16·27 grams

**Imputing Climate Change Agreement to NHANES data**

To impute an attitude on climate change to NHANES respondents, a logistic regression model was developed with the US subsample (n=1,051) of the Chatham House data using a dichotomous dependent variable (i.e. agrees or not that humans contribute to climate change) and all independent variables that were also available in NHANES: age, gender, education, household size, and income-to-poverty ratio. Coefficients from this model (Appendix Table 4) and observed demographic characteristics from NHANES respondents were used to calculate NHANES individuals' predicted probabilities of agreement that humans contribute to climate change (see Appendix Figure 1).

**Appendix Table 4. Coefficients used to predict agreement that humans contribute to climate change in Chatham House Survey***

| | **Coefficient** | **Standard Error** | **P>\|z\|** |
|---|---|---|---|
| **Female** | 0·483 | 0·240 | 0·04 |
| **Age** | | | |
| 18-29 years | | | |
| 30-49 years | 0·436 | 0·405 | 0·28 |
| 40-49 years | -0·524 | 0·420 | 0·21 |
| 50-65 years | -0·457 | 0·355 | 0·20 |
| **Education** | | | |
| Less than high school | | | |
| High school grad/GED | 0·319 | 0·536 | 0·55 |
| Some college | 0·491 | 0·529 | 0·35 |
| College grad or higher | 0·272 | 0·533 | 0·61 |
| **Income-to-Poverty Ratio** | | | |
| 1 - < 2 | | | |
| 2 - < 5 | 0·531 | 0·330 | 0·11 |
| 5 - < 10 | 0·415 | 0·382 | 0·28 |
| >=10 | 0·376 | 0·713 | 0·60 |
| **Household Size** | -0·227 | 0·103 | 0·03 |
| Intercept | 0·740 | 0·572 | 0·20 |
| N | 939 | | |
| Wald chi-squared | 22·56 | | |
| P > chi-squared | 0·02 | | |
| Pseudo $R^2$ | 0·063 | | |

*The model was a multi-variable logistic regression run on all US survey participants with complete sociodemographic data (939 of 1,051) between 18 and 65 years old. The outcome variable was equal to 1 if the respondent agreed (strongly agreed or tended to agree) that humans contribute to climate change and equal to 0 otherwise. All independent variables in the model are shown in the table.

These predicted probabilities were categorized into a dichotomous variable equal to 1 if the probability was greater than 0·615. We used this cut point to create the same proportion of agreeing individuals in NHANES as in Chatham House (69%, n=715), since both are nationally representative samples of the US adult population. The distribution of sociodemographic characteristics differs between the Chatham House dataset and NHANES. Therefore, before imputation, the Chatham House data were reweighted using entropy balancing weights. This multivariate reweighting method calibrates unit weights for Chatham House to balance covariate means between it and the NHANES dataset (Appendix Table 5).[2]

**Appendix Table 5. Entropy balancing process: Covariate means with original dataset sample weights and with entropy weights**

| | NHANES: Survey design and sample weights included | | | Chatham House: Sample weights | | | Chatham House: Entropy balanced weights | | |
|---|---|---|---|---|---|---|---|---|---|
| | **Mean** | **Variance** | **Skewness** | **Mean** | **Variance** | **Skewness** | **Mean** | **Variance** | **Skewness** |
| Age [18,30) | 0·260 | 0·192 | 1·096 | 0·225 | 0·175 | 1·317 | 0·260 | 0·192 | 1·096 |
| Age [30,40) | 0·230 | 0·177 | 1·285 | 0·166 | 0·139 | 1·796 | 0·230 | 0·177 | 1·285 |
| Age [40,50) | 0·306 | 0·212 | 0·843 | 0·394 | 0·239 | 0·435 | 0·306 | 0·213 | 0·843 |
| Household size | 3·201 | 2·359 | 0·610 | 2·587 | 1·816 | 0·898 | 3·201 | 2·847 | 0·572 |
| Male | 0·487 | 0·250 | 0·051 | 0·494 | 0·250 | 0·026 | 0·487 | 0·250 | 0·051 |
| Less than high school | 0·243 | 0·184 | 1·200 | 0·182 | 0·149 | 1·652 | 0·243 | 0·184 | 1·201 |
| High School grad/GED | 0·311 | 0·214 | 0·815 | 0·375 | 0·235 | 0·515 | 0·311 | 0·215 | 0·815 |
| Some College | 0·263 | 0·194 | 1·077 | 0·417 | 0·243 | 0·337 | 0·263 | 0·194 | 1·076 |
| Employed | 0·701 | 0·210 | -0·878 | 0·608 | 0·239 | -0·442 | 0·701 | 0·210 | -0·877 |
| Unemployed | 0·129 | 0·113 | 2·210 | 0·101 | 0·091 | 2·652 | 0·129 | 0·113 | 2·210 |
| Student | 0·041 | 0·040 | 4·614 | 0·107 | 0·096 | 2·537 | 0·041 | 0·040 | 4·603 |
| Retired | 0·071 | 0·066 | 3·345 | 0·114 | 0·101 | 2·434 | 0·071 | 0·066 | 3·343 |
| Homemaker | 0·029 | 0·028 | 5·607 | 0·010 | 0·010 | 10·050 | 0·029 | 0·028 | 5·609 |
| Income-to-Poverty Ratio (0,1] | 0·129 | 0·112 | 2·218 | 0·142 | 0·122 | 2·047 | 0·129 | 0·112 | 2·216 |
| Income-to-Poverty Ratio (1,2] | 0·187 | 0·152 | 1·606 | 0·214 | 0·168 | 1·396 | 0·187 | 0·152 | 1·606 |
| Income-to-Poverty Ratio (2,5] | 0·369 | 0·233 | 0·544 | 0·434 | 0·246 | 0·266 | 0·369 | 0·233 | 0·544 |
| Income-to-Poverty Ratio (5,10] | 0·238 | 0·182 | 1·228 | 0·198 | 0·159 | 1·514 | 0·238 | 0·182 | 1·229 |

**Appendix Table 6. Coefficients used to predict Health Eating Index and diet cost in NHANES[1]**

| | Model to predict Health Eating Index | | | Model to predict diet cost | | |
|---|---|---|---|---|---|---|
| | **Coef.** | **Std. Err.** | **P>\|z\|** | **Coef.** | **Std. Err.** | **P>\|z\|** |
| Crop Group: Beef | -0·015 | 0·002 | <0·0001 | 0·001 | 0·000 | <0·0001 |
| Crop Group: Other ruminant meat | -0·023 | 0·008 | 0·02 | 0·003 | 0·001 | 0.00 |
| Crop Group: Pork | -0·021 | 0·004 | <0·0001 | 0·001 | 0·000 | 0.01 |
| Crop Group: Poultry | 0·007 | 0·002 | <0·0001 | 0·001 | 0·000 | <0·0001 |
| Crop Group: Other nonruminant meat | 0·282 | 0·020 | <0·0001 | 0·006 | 0·001 | <0·0001 |
| Crop Group: Fish & Seafood | 0·024 | 0·003 | <0·0001 | 0·005 | 0·000 | <0·0001 |
| Crop Group: Eggs | -0·009 | 0·004 | 0·03 | -0·001 | 0·000 | <0·0001 |
| Crop Group: Dairy | 0·006 | 0·001 | <0·0001 | 0·000 | 0·000 | <0·0001 |
| Crop Group: Oils | -0·022 | 0·008 | 0·01 | -0·011 | 0·001 | <0·0001 |
| Crop Group: Solid (plant) fats | -0·016 | 0·069 | 0·84 | 0·006 | 0·003 | 0.05 |
| Crop Group: Vegetables & Juices | 0·011 | 0·004 | <0·0001 | 0·001 | 0·000 | <0·0001 |
| Crop Group: Fruits & Juices | 0·016 | 0·001 | <0·0001 | 0·000 | 0·000 | <0·0001 |
| Crop Group: Legumes | 0·051 | 0·009 | <0·0001 | -0·002 | 0·000 | <0·0001 |
| Crop Group: Nuts & Seeds | 0·096 | 0·017 | <0·0001 | -0·002 | 0·001 | 0.01 |
| Crop Group: Soy | 0·027 | 0·005 | <0·0001 | 0·001 | 0·000 | <0·0001 |
| Crop Group: Grains | -0·010 | 0·002 | <0·0001 | 0·001 | 0·000 | <0·0001 |
| Crop Group: Beverages | 0·000 | 0·000 | 0.01 | 0·000 | 0·000 | 0.01 |
| Crop Group: Sweeteners | -0·040 | 0·002 | <0·0001 | -0·002 | 0·000 | <0·0001 |
| Crop Group: Other | 0·014 | 0·018 | 0.42 | 0·008 | 0·001 | <0·0001 |
| Women | 1·146 | 0·407 | 0.00 | 0·103 | 0·022 | <0·0001 |
| Household size | 0·143 | 0·127 | 0.30 | -0·015 | 0·008 | 0.08 |
| Age [30,40) | 0·662 | 0·527 | 0.18 | 0·048 | 0·038 | 0.20 |
| Age [40,50) | 1·276 | 0·525 | 0.04 | 0·068 | 0·042 | 0.06 |
| Age [50,65) | 3·264 | 0·558 | <0·0001 | 0·101 | 0·044 | 0.01 |
| High School grad/GED | 0·737 | 0·569 | 0.29 | 0·047 | 0·043 | 0.20 |
| Some College | 2·043 | 0·571 | <0·0001 | 0·073 | 0·031 | 0.05 |
| College | 4·134 | 0·674 | <0·0001 | 0·098 | 0·040 | 0.02 |
| Income-to-Poverty Ratio (2,5] | 0·852 | 0·429 | 0.04 | 0·065 | 0·028 | 0.03 |
| Income-to-Poverty Ratio (5,10] | 1·011 | 0·563 | 0.18 | 0·108 | 0·036 | 0.00 |
| Income-to-Poverty Ratio>10 | 1·501 | 0·926 | 0.12 | 0·204 | 0·044 | <0·0001 |
| Hispanic | 0·719 | 0·463 | 0.32 | -0·166 | 0·032 | <0·0001 |
| Black | -0·774 | 0·432 | 0.11 | -0·137 | 0·035 | <0·0001 |
| Other race | -0·032 | 0·817 | 0.97 | -0·219 | 0·050 | <0·0001 |
| Intercept | 42·044 | 1·036 | <0·0001 | 2·358 | 0·060 | <0·0001 |
| N | 7,188 | | | 7,188 | | |
| $R^2$ | 0·4371 | | | 0·3183 | | |

***** Coefficient estimates in this table are the results of two separate multi-variable regressions models, one with Healthy Eating Index as the dependent variable, and one with Diet Cost as the dependent variable. All variables in the respective models are included in the table.

**Appendix Table 7: Mean grams of commodity intake by potential changers under various meat replacement scenarios***

| | **Beef** | | **Pork** | | **Poultry** | | **Legumes** | | **Nuts/Seeds** | |
|---|---|---|---|---|---|---|---|---|---|---|
| | Mean | 95% CI | Mean | 95% CI | Mean | 95% CI | Mean | 95% CI | Mean | 95% CI |
| **Baseline diet** | 37·7 | (32·3, 43·0) | 20·1 | (15·8, 24·5) | 53·8 | (49·9, 57·7) | 19·4 | (14·7, 24·1) | 10·3 | (8·8, 11·8) |
| **100% beef replaced:** | | | | | | | | | | |
| With poultry | 0·0 | ·· | ·· | ·· | 96·0 | (90·6, 101·4) | ·· | ·· | ·· | ·· |
| With plant protein[3] | 0·0 | ·· | ·· | ·· | ·· | ·· | 44·5 | (36·5, 52·6) | 15·8 | (14·3, 17·3) |
| **50% beef replaced:** | | | | | | | 32·0 | (26·1, 37·8) | 13·1 | (11·6, 14·5) |
| With poultry | 18·8 | (16·2, 21·5) | ·· | | 74·9 | (71·2, 78·6) | ·· | ·· | ·· | ·· |
| With plant protein[3] | 18·8 | (16·2, 21·5) | ·· | | ·· | ·· | ·· | ·· | ·· | ·· |
| **25% beef replaced:** | | | | | | | | | | |
| With poultry | 28·2 | (24·2, 32·3) | ·· | | 64·4 | (60·9, 67·8) | ·· | ·· | ·· | ·· |
| With plant protein[3] | 28·2 | (24·2, 32·3) | ·· | | ·· | ·· | 25·7 | (20·6, 30·7) | 11·7 | (10·3, 13·1) |
| **100% beef, pork, poultry replaced:** | | | | | | | | | | |
| With plant protein[3] | 0·0 | ·· | 0·0 | ·· | 0·0 | ·· | 96·4 | (80·7, 112·1) | 24·8 | (22·6, 27·0) |
| **50% beef, pork, poultry replaced:** | | | | | | | | | | |
| With plant protein[3] | 18·8 | (16·2, 21·5) | 10·1 | (7·9, 12·2) | 26·9 | (24·9, 28·9) | 57·9 | (48·5, 67·3) | 17·6 | (15·9, 19·3) |
| **25% beef, pork, poultry replaced:** | | | | | | | | | | |
| With plant protein[3] | 28·2 | (24·2, 32·3) | 15·1 | (11·8, 18·4) | 40·4 | (37·4, 43·3) | 38·6 | (32·0, 45·2) | 14·0 | (12·4, 15·5) |

***** Commodity amounts of edible portion of the different meats and plant foods in grams per day. Blank cells under replacement scenarios represent no change in commodity consumption from baseline.

**Appendix Figure 1. Predicted probabilities to agree that humans contribute to climate change in Chatham House and in NHANES***

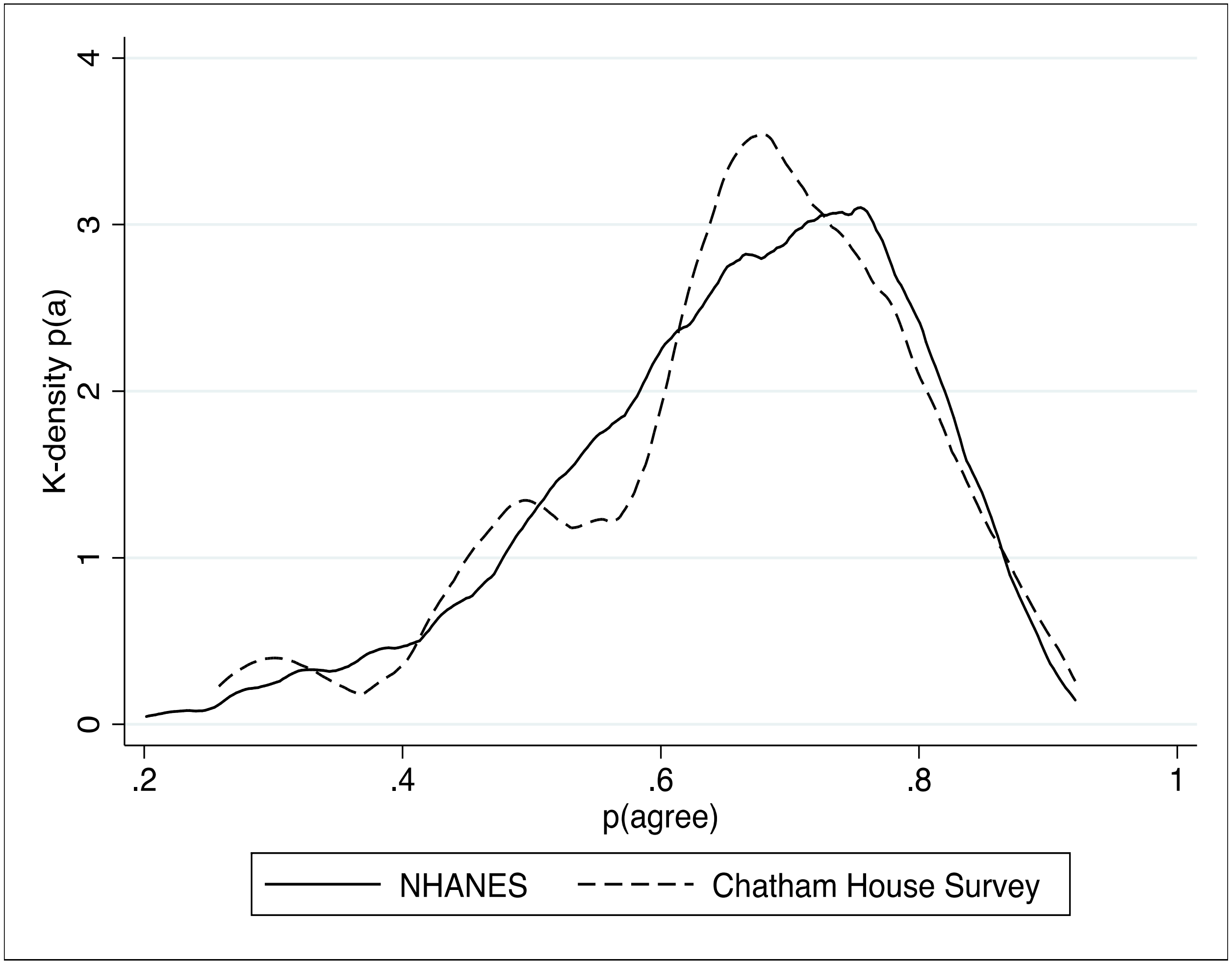


***Predicted probabilities of agreement [p(agree)] are calculated for all individuals using coefficients of the logistic regression model depicted in Appendix Table 4 and observed values of sociodemographic variables. This figure depicts the distribution of probabilities in the original Chatham House data compared to the distribution of probabilities imputed to NHANES respondents.

Appendix References

1 Guenther PM, Casavale KO, Reedy J, et al. Update of the Healthy Eating Index: HEI-2010. *J Acad Nutr Diet* 2013; **113**(4): 569–80.
2 Hainmueller J. Entropy Balancing for Causal Effects: A Multivariate Reweighting Method to Produce Balanced Samples in Observational Studies. *Political Analysis* 2012; **20**(1): 25–46.
